# AI-driven lateral flow immunoassay for point-of-care detection of cardiac biomarkers in acute myocardial infarction

**DOI:** 10.1007/s00604-026-08054-y

**Published:** 2026-04-20

**Authors:** Yuhan Lu, Xiaoyun Peng, Zhao Yin, Xiaoqin Fan, Jingchun Fan, Yali Mi, Guangchao Li

**Affiliations:** 1 College of Life Sciences and Technology, Jinan University, Guangzhou, No. 601 Huangpu Avenue West, Tianhe District, 510632 China; 2 Krieger School of Arts & Sciences, Johns Hopkins University, Baltimore, MD 21218 USA; 3 Purecells Medical Technology (Guangzhou) Co., Ltd, Guangzhou, Guangdong 510700 China; 4 Department of Hematology, The Affiliated Guangdong Second Provincial General Hospital of Jinan University, Guangzhou, Guangdong 510317 China; 5 The Biobank of Shenzhen Second People’s Hospital, The First Affiliated Hospital of Shenzhen University, Shenzhen, Guangdong No. 3002, Sungang West Road, Futian District, 518035 China; 6 School of pharmaceutical sciences, Guangzhou Medical University, Guangzhou, No. 1, Xinzhao Road, Xinzhao Town, Panyu District, 511436 China

**Keywords:** Gold nanoparticle, Immunoassay, Point-of-care detection, Cardiac biomarkers, Acute myocardial infarction

## Abstract

A functionalized gold nanoparticle-based lateral flow immunoassay (AuNPs-LFIA) platform integrated with a deep learning-based machine vision model for the rapid and quantitative detection of cardiac biomarkers, myoglobin (Myo) and cardiac troponin I (cTnI). AuNPs@Ab probes with high specificity and stability were prepared. AuNPs were synthesized via the citrate reduction method, functionalized with HS-PEG-COOH, and covalently conjugated to antibodies through EDC/NHS-mediated coupling to yield high specificity and stability. Using this platform, the machine vision-assisted AuNPs-LFIA achieved detection limits of 0.224 ng/mL for Myo and 0.071 ng/mL for cTnI. Quantitative readouts were obtained within 8 min, representing a 46.7% improvement in detection efficiency compared to conventional methods. Analysis of spiked serum samples demonstrated a significant correlation with commercial assay kits (R² > 0.99), further validating the platform’s accuracy and clinical applicability. Overall, this AI-enhanced AuNPs-LFIA platform offers sensitive, rapid, and user-friendly quantification. Its robust scalability provides a practical strategy for point-of-care testing (POCT) in cardiovascular diagnostics, particularly in resource-limited settings.

**Graphical Abstract:**

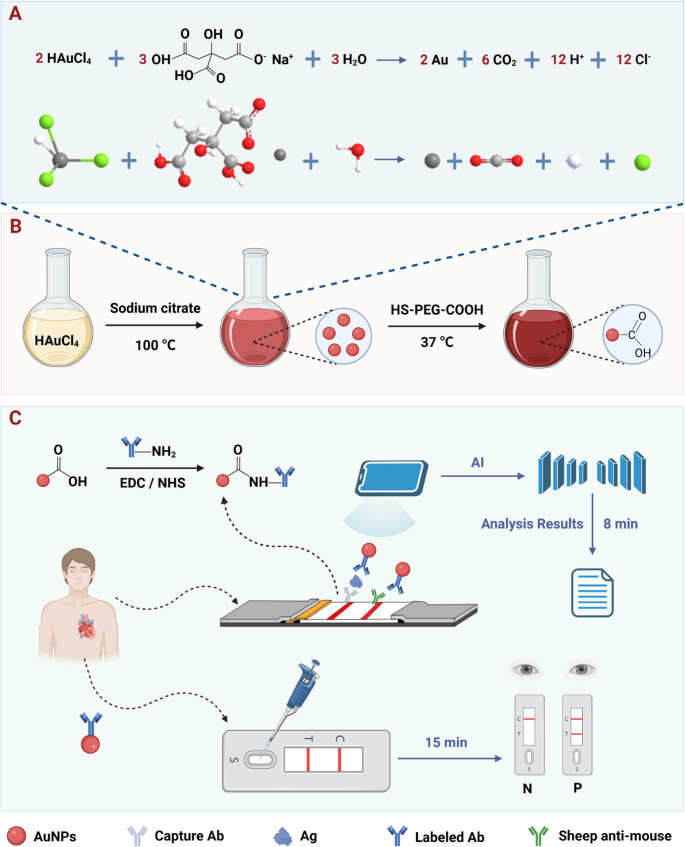

**Supplementary Information:**

The online version contains supplementary material available at 10.1007/s00604-026-08054-y.

## Introduction

Achieving high sensitivity and rapid quantitative diagnosis remains a major challenge in point-of-care testing (POCT) for acute myocardial infarction (AMI), where clinicians require biomarker results within the critical “golden hour” to guide thrombolytic therapy. While numerous POCT platforms exist, each presents distinct limitations that hinder emergency deployment [1, 2]. Immunoassays, such as enzyme-linked immunosorbent assay (ELISA), similarly demand 3–5 h to complete [3–5]. In contrast, paper-based lateral flow immunoassays (LFIA) deliver qualitative results within approximately 15 min, offering a substantial time advantage [6, 7]. However, in emergency medicine, particularly for acute myocardial infarction (AMI)-a prevalent and highly fatal cardiovascular disease-clinicians demand both rapid and quantitatively reliable readouts [8, 9]. Early AMI diagnosis further benefits from the simultaneous detection of multiple cardiac biomarkers, with myoglobin (Myo) and cardiac troponin I (cTnI) recognized as critical indicators [10–14]. Their complementary sensitivity and specificity provide a stronger basis for clinical decision-making.

Molecular diagnostics (RT-PCR, LAMP) provide gold-standard analytical accuracy (10²-10³ copies/mL sensitivity) but require 1–4 h of sample processing, thermal cycling (60–95 °C), and skilled operators, rendering them impractical for ambulance or rural clinic use. Laboratory-based immunoassays like ELISA achieve pg/mL limits of detection with excellent quantification but demand 3–5 h, multi-step washing protocols, and 96-well infrastructure. Electrochemical sensors offer portability and multiplexing (10 pg/mL LODs) yet require custom electrode functionalization, potentiostats, and frequent recalibration.

Current point-of-care testing (POCT) platforms for cardiac biomarker detection vary widely in performance, with each technology presenting specific limitations for emergency use. Molecular methods like RT-PCR and LAMP achieve high analytical sensitivity but require 60–240 min, thermal cycling, and expert operators at $10–50 per test, precluding field deployment. ELISA provides pg/mL quantification through 180-300-minute multi-step protocols needing lab infrastructure. Electrochemical sensors offer portable multiplexing but demand electrode preparation and calibration equipment. Conventional LFIA excels in speed and cost with full field-deployability, yet remains qualitative with 5–50 ng/mL LODs and 20–30% visual interpretation variability.

Conventional LFIA represent the current POCT standard due to their 15-minute speed, <$1 cost, and minimal training requirements. However, they deliver only qualitative “yes/no” results via visual interpretation, suffer 20–30% inter-operator variability, and exhibit poor analytical sensitivity (~ 5–50 ng/mL LODs for cardiac biomarkers). Smartphone LFIA readers improve semi-quantitative accuracy (R²~0.95) but still mandate full 15-minute color development plus mobile app processing.

This study addresses these gaps through synergistic innovations that no prior platform combines: (1) covalently-functionalized AuNPs@Ab probes delivering 22–70× lower LODs than visual LFIA; (2) YOLOv11 machine vision enabling automated 8-minute readouts by detecting partially-developed T/C lines invisible to the human eye; and (3) embedded edge computing that eliminates operator subjectivity. This unique combination establishes the first truly operator-independent, field-deployable quantitative LFIA for AMI triage.

Early diagnosis particularly benefits from simultaneous Myo/cTnI detection, leveraging their complementary release kinetics (Myo rises first for ultra-early detection; cTnI peaks later for confirmation). While AuNPs serve as established LFIA labels, their typical physisorption compromises probe stability during capillary flow. Our HS-PEG-COOH covalent conjugation strategy, combined with AI integration, overcomes these longstanding limitations to create a practical POCT standard for resource-constrained environments.

Commonly employed LFIA probes include fluorescent microspheres, quantum dots, and colloidal gold (AuNPs). While fluorescent probes can enhance sensitivity, they are prone to photobleaching and require specialized detection equipment, increasing cost and compromising portability-limitations that hinder their use in resource-limited or decentralized POCT settings [15–18]. By contrast, AuNPs are widely used due to their intuitive colorimetric readout and operational simplicity. Yet, conventional AuNP-based probes face three primary limitations: (i) antibody (Ab) is typically adsorbed via noncovalent interactions, reducing probe stability and specificity; (ii) results rely on visual interpretation, introducing subjectivity; and (iii) quantitative accuracy remains limited [19–21]. These shortcomings highlight the need for functionalized probes with enhanced stability and specificity, coupled with rapid and reliable quantitative readout strategies.

**Fig. 1:**
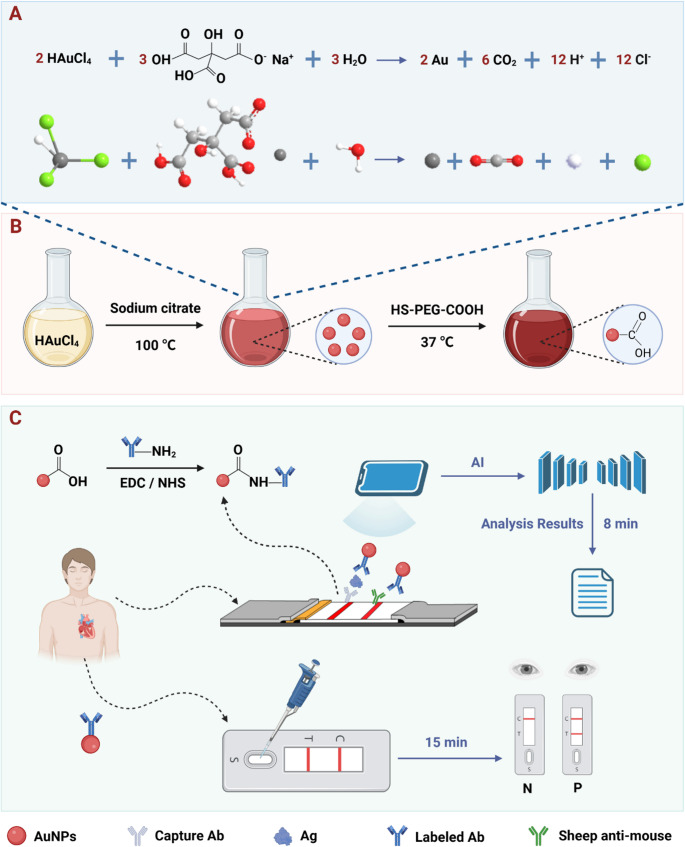
Workflow overview of AI-enhanced LFIA for AMI biomarkers. (**A**) AuNP synthesis via citrate reduction. (**B**) Covalent AuNPs@Ab conjugation (HS-PEG-COOH → EDC/NHS → Ab). (**C**) Platform comparison: AI-LFIA achieves 8-min quantitative readout (YOLOv11 T/C analysis) vs. commercial LFIA 15-min visual inspection, with 22–70× lower LODs and < 1.4% operator variability

Recent advances in machine vision offer a promising solution for rapid, quantitative LFIA [22–27]. Unlike traditional hardware-based optimization, machine vision leverages image recognition and pattern learning to accelerate detection, enhance accuracy, and improve robustness [28–30]. Motivated by this potential, we developed an LFIA platform integrated with a deep learning-based machine vision framework for the rapid and quantitative detection of Myo and cTnI, utilizing functionalized AuNPs@Ab probes with enhanced stability and specificity. Motivated by this potential, we developed an LFIA platform integrated with a deep learning-based machine vision framework for the rapid quantitative detection of Myo and cTnI, utilizing covalently-functionalized AuNPs@Ab probes (Fig. 1A-C). Figure 1C illustrates the platform’s workflow advantage over commercial LFIA: 8-min AI-quantitative readout vs. 15-min visual inspection. This integration reduces assay time by 46.7% while eliminating subjective interpretation errors. The core innovation lies in the machine vision system, which enables automated quantitative readout in just 8 min while minimizing subjective errors. Collectively, this work establishes a rapid and practical strategy for the early detection of myocardial injury, highlighting the potential of AI-enhanced LFIA for point-of-care quantitative diagnostics.

## Materials and methods

### Materials and reagents

Chloroauric acid hydrated (HAuCl_4_) was from Beijing Xinsheng Baitai Technology Co., LTD (Shanghai, China). Sodium citrate, bovine serum albumin (BSA), fetal bovine serum (FBS), sucrose and sodium chloride were from Shanghai Maclean Biochemical Technology Co., LTD (Maclin, Shanghai). Thiol-polyethylene glycol-carboxylic acid was from Guangzhou Jiexin Biotechnology Co., LTD. Nitrocellulose (NC) membranes, glass fiber and absorbent pad were from Shanghai, China. Myo 212 (capture Ab), Myo 213 (labeled Ab), Myo antigen, cTnI monoclonal antibody (capture Ab), cTnI monoclonal antibody (labeled Ab), cTnI antigen and goat anti-mouse IgG (H + L) were from Qianyuan Shunze Technology Co., LTD (Qingdao, China). The kits of Myo and cTnI were from Taobao.

### Synthesis of gold nanoparticles

Gold nanoparticles (AuNPs) with an average diameter of approximately 20 nm were synthesized using the classical citrate reduction method [31]. Briefly, 13 µL of 1 mM chloroauric acid (HAuCl₄) solution was added to 50 mL of ultrapure water in a 100 mL round-bottom flask and brought to a boil under magnetic stirring. In parallel, a 1% (w/v) sodium citrate solution was prepared by dissolving 0.03 g of sodium citrate in 3 mL of ultrapure water. Once the HAuCl₄ solution reached boiling, 2 mL of the sodium citrate solution was rapidly added. During the reaction, the color of the solution changed sequentially from pale yellow to gray, purple, and finally to wine red, indicating the successful formation of AuNPs. The mixture was maintained at boiling for an additional 15 min and then allowed to cool naturally to room temperature. The resulting AuNPs were stored at 4 °C for further use. To improve the colloidal stability and introduce functional groups, the surface of the AuNPs was modified with thiol-terminated polyethylene glycol bearing a carboxyl group (HS-PEG-COOH). A 5 mg/mL HS-PEG-COOH aqueous solution was prepared, and 200 µL of this solution was added to 25 mL of the synthesized AuNPs. The mixture was incubated on a shaker (320 rpm) at room temperature for 2 h. Subsequently, the solution was left undisturbed at 4 °C overnight to promote the formation of stable Au-S bonds between the thiol groups and the AuNP surfaces. Unbound HS-PEG-COOH molecules were removed by repeated washing with ultrapure water, and the carboxyl-functionalized AuNPs were finally resuspended in 25 mL of ultrapure water and stored at 4 °C for future use.

### Covalent conjugation of antibodies to gold nanoparticles

Carboxyl-functionalized AuNPs were conjugated with antibodies via a carbodiimide-mediated amide coupling strategy. Briefly, 1 mL of carboxylated AuNP solution was incubated with 10 µL of 0.4 mM EDC (1-ethyl-3-(3-dimethylaminopropyl) carbodiimide hydrochloride) and 0.1 mM NHS (N-hydroxysuccinimide) in MES buffer (pH 6.0) at room temperature with gentle shaking for 15 min to activate the surface carboxyl groups into reactive NHS ester intermediates. Following activation, the mixture was centrifuged at 10,000 rpm for 6 min to remove excess EDC and NHS, and the pellet was resuspended in ultrapure water. Next, 15 µg of antibody was added to the activated AuNPs and incubated at room temperature for 1 h to facilitate covalent coupling between the NHS esters and the primary amine groups on the antibody via stable amide bond formation. Afterward, 100 µL of 10% (w/v) bovine serum albumin (BSA) was added and incubated for an additional 30 min to block non-specific binding sites. Finally, the conjugates were centrifuged at 12,000 rpm for 6 min, and the supernatant was discarded. The resulting AuNPs@Ab conjugates were resuspended in 50 µL of running buffer for storage and future use.

### Preparation of LFA test strips

The LFA strip was composed of five components: a sample pad, a NC membrane, an absorbent pad, a conjugate pad (glass fiber), and a polyvinyl chloride (PVC) backing card. Glass fiber was selected as the sample pad material and pretreated by immersing it in a borate buffer solution (pH 7.5) containing 5% bovine serum albumin (BSA), 2% sucrose, and 0.5% Tween-20, followed by air drying at room temperature. Subsequently, the sample pad, conjugate pad, NC membrane, and absorbent pad were sequentially assembled onto the PVC backing, with each pad overlapping the adjacent one by 3 mm. The capture antibodies for myoglobin (Myo) and cardiac troponin I (cTnI), as well as the goat anti-mouse IgG antibody (control line), were diluted in phosphate-buffered saline (PBS) to final concentrations of 0.8 mg/mL, 1 mg/mL, and 0.5 mg/mL, respectively. Using a dispenser, the antibodies were dispensed onto the NC membrane at a rate of 0.5 µL/cm to form the test lines (T-lines) and control lines (C-lines). After dispensing, the NC membrane was dried at 37 °C for 2 h. Finally, the assembled sheet was cut into 3 mm-wide individual strips using a cutter. The test strips were sealed in foil pouches containing desiccant and stored at room temperature until further use.

### Optimization of immunoassay parameters for enhanced sensitivity

To achieve optimal detection sensitivity and ensure the reliability of the platform for practical applications, several critical parameters in the LFIA were systematically optimized. The parameters optimized included the volume of AuNPs@Ab conjugates, the sample volume, and the concentration of capture antibody at the test line. A one-variable-at-a-time strategy was applied, with the T/C intensity ratio at a fixed antigen concentration serving as the evaluation criterion for the comparative metric. Using myoglobin (Myo) as a model analyte, the results demonstrated that optimal performance was achieved with 1 µL of AuNPs@Ab, a 60 µL sample volume, and a capture antibody concentration of 1 mg/mL. For cardiac troponin I (cTnI), the optimal condition was obtained using 1 µL of AuNPs@Ab, a 50 µL sample volume, and a capture antibody concentration of 0.8 mg/mL.

AI Dataset and Machine Vision System The machine vision system utilized a CCD industrial camera (Basler ace acA1920-155 μm, 12 MP resolution, 155 fps) mounted on a custom enclosure for consistent imaging. Images were captured at 1920 × 1080 resolution under diffuse LED illumination (5000 K, 300 lx) and processed by an embedded NVIDIA Jetson Orin Nano (8GB RAM, 1024 CUDA cores) running Ubuntu 20.04 with CUDA 12.2.

The detection model was implemented using YOLOv11 via the Ultralytics framework (v8.3.0). The dataset comprised 750 images (total 4,500 strip instances) captured from experimental assays, featuring variations in strip position (1–4 per image), orientation (± 30°), illumination (200–800 lx), and backgrounds (white, wood, fabric). Images were manually annotated using LabelImg v1.8.6 with two classes: “T-line” and “C-line.” The dataset was split 80/13/7% (600 train, 100 val, 50 test images).

Training parameters: SGD optimizer (momentum = 0.937, weight_decay = 0.0005), initial lr = 0.01 with cosine annealing to 0.001 over 450 epochs, batch_size = 16, img_size = 640. Composite loss: VarifocalLoss (classification) + CIoU Loss (bounding boxes) + DFL Loss (distribution focal). Data augmentation: Mosaic(1.0), MixUp(0.1), random rotation(± 15°), brightness/contrast(± 0.2), HSV hue/sat/val(± 0.015/0.7/0.4), Gaussian noise(σ = 0.01). Early stopping (patience = 50 epochs) yielded final mAP@0.5 = 0.923 (val), mAP@0.5:0.95 = 0.784. The inference time averaged 12 ms per image (83 fps).

### Data processing

To evaluate the performance of the proposed AI-based detection model, standard classification metrics were employed, including accuracy, recall (sensitivity), and precision.

Accuracy represents the overall proportion of correctly classified instances among all predictions and is defined as:

Accuracy = (TP + TN) / (TP + TN + FP + FN).

Recall (Sensitivity or True Positive Rate) measures the ability of the model to correctly identify positive instances and is given by:

Recall = TP / (TP + FN).

Precision (Positive Predictive Value) indicates the proportion of predicted positive instances that are truly positive and is defined as:

Precision = TP / (TP + FP).

In these expressions, TP (true positives) denotes correctly detected target signals, TN (true negatives) represents correctly identified non-target instances, FP (false positives) corresponds to incorrectly detected positives, and FN (false negatives) indicates missed detections.

These metrics were calculated to comprehensively assess the detection performance and reliability of the proposed model under varying experimental conditions.

### Spiked sample analysis

To evaluate the quantitative performance of the constructed AuNPs-LFIA platform in practical samples, fetal bovine serum (FBS) was used as the matrix, and different concentrations of myoglobin (Myo) and cardiac troponin I (cTnI) were spiked to simulate clinical samples. Each spiked sample was tested in triplicate. For LFA detection, an image recognition approach incorporating the YOLO object detection algorithm was employed to capture and analyze the test strips. Images of the strips were acquired using a CCD, and the trained YOLO model automatically identified the test line (T line) and control line (C line), extracting their R-channel values to calculate the T/C ratio, which was then used for the quantitative determination of Myo or cTnI concentrations. All test strips were imaged under the same platform to ensure data consistency. For validation, commercial Myo and cTnI kits were used to quantify the concentrations of the respective biomarkers in the spiked FBS samples according the manufacturer’s protocols strictly.

## Results and discussion

### Characterization of AuNPs and AuNPs@Ab

To construct antibody (Ab)-functionalized gold nanoparticles (AuNPs@Ab) for colorimetric detection, AuNPs were synthesized via the classical sodium citrate reduction method. In this approach, chloroauric acid (HAuCl₄) served as the gold precursor, and sodium citrate acted as both reducing and stabilizing agent. Under heating conditions, Au³⁺ ions were reduced to elemental gold, yielding monodisperse AuNPs with uniform particle size (Fig. 1A). To introduce reactive functional groups onto the AuNP surface for subsequent covalent Ab conjugation, the nanoparticles were modified with thiol-polyethylene glycol-carboxyl (HS-PEG-COOH). This modification exploits the strong covalent Au-S bond between the thiol group and the gold surface, ensuring stable PEG attachment while presenting terminal carboxyl groups as active sites for Ab coupling (Fig. 1B).

**Fig. 2:**
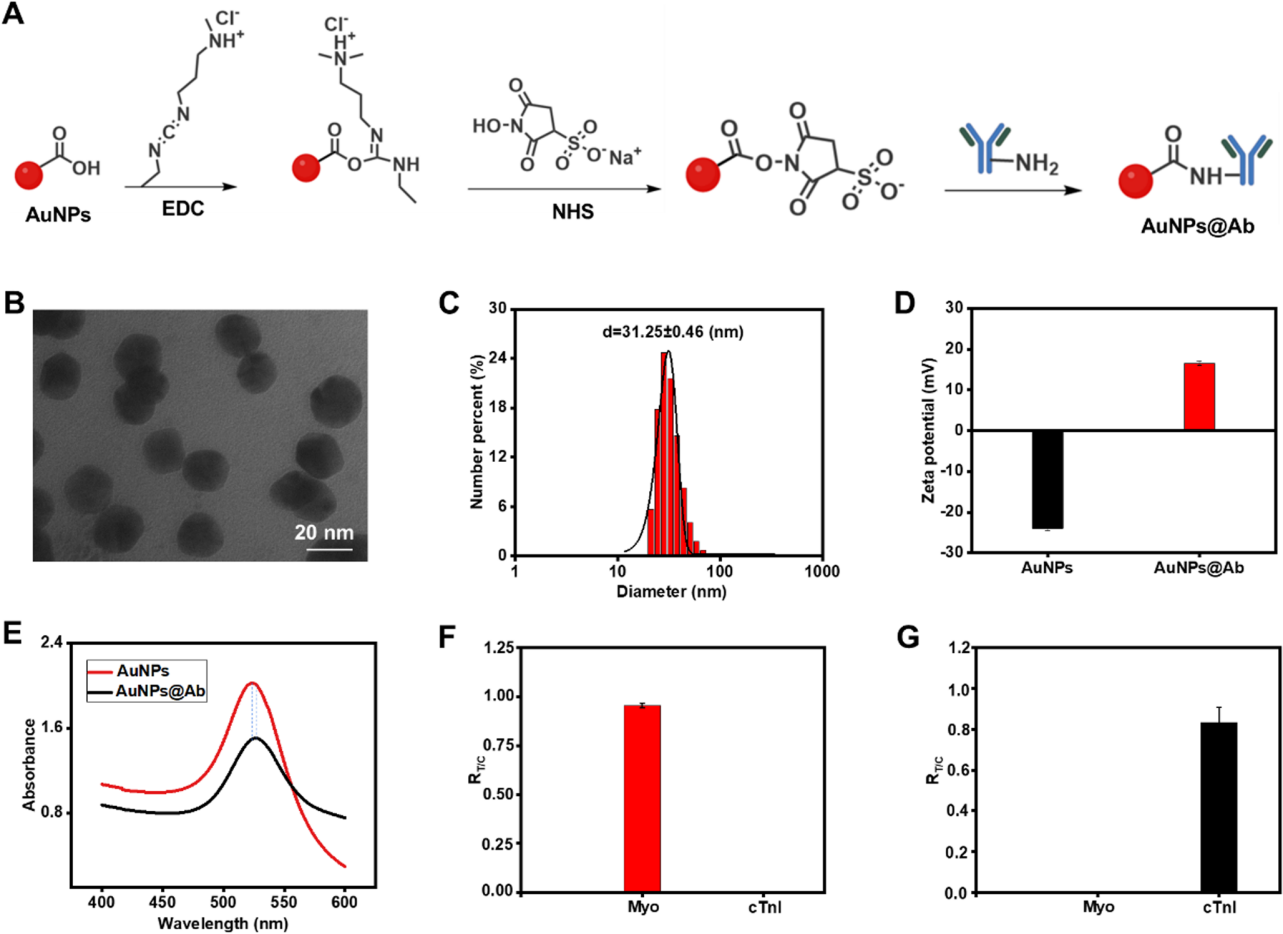
Characterization of AuNPs and AuNPs@Ab. (**A**) Schematic diagram of the process for the covalent coupling Ab of AuNPs. (**B**) TEM characterization of AuNPs. (**C**) Dynamic light scattering characterization of AuNPs. (**D**) Zeta sites characterize AuNPs and AuNPs@Ab. (**E**) Ultraviolet absorption spectra of AuNPs and AuNPs@Ab. (**F**) Specificity analysis of Myo. (**G**) Specificity analysis of cTnI

Following surface modification, the carboxyl groups were activated with 1-ethyl-3-(3-dimethylaminopropyl) carbodiimide (EDC) and N-hydroxysuccinimide (NHS) to facilitate amide bond formation between the activated carboxyl groups and primary amines of lysine residues in the target antibody. This reaction resulted in the covalent immobilization of antibodies on the AuNP surface (Fig. 2A). To characterize the morphology, size, and surface properties of the synthesized AuNPs and AuNPs@Ab probes, multiple analytical techniques were employed, including transmission electron microscopy (TEM), dynamic light scattering (DLS), zeta potential measurements, and UV-vis spectroscopy. TEM imaging revealed that the synthesized AuNPs were highly dispersed spherical particles with an average diameter of approximately 20 nm (Fig. 2B), indicating uniform size distribution and favorable dispersibility, advantageous for stable migration through LFIA strips. DLS analysis showed a hydrodynamic diameter of 31.25 ± 0.46 nm with a narrow size distribution, confirming monodispersity in aqueous solution (Fig. 2C). Upon Ab conjugation, the zeta potential shifted significantly from − 24.0 ± 0.53 mV to + 16.6 ± 0.55 mV (Fig. 2D), indicating a substantial alteration in surface charge and successful covalent attachment of antibodies to the AuNP surface. Moreover, the UV-vis absorption peak of AuNPs exhibited a slight red shift from 524 nm to 527 nm after Ab conjugation, further corroborating effective antibody immobilization (Fig. 2E).

To evaluate antibody specificity, cross-reactivity assays were conducted using AuNPs@Myo-Ab with cTnI antigen and AuNPs@cTnI-Ab with Myo antigen. No detectable T-line signal was observed in either mismatched configuration (Figs. 2F, G), confirming high target selectivity and absence of inter-biomarker cross-reactivity.

### Interference and matrix specificity analysis

To assess robustness against common serum interferents, we systematically evaluated platform performance in FBS spiked with clinically relevant concentrations of potential interfering substances: hemoglobin (10 mg/dL, hemolysis simulation), bilirubin (20 mg/dL, jaundice simulation), triglycerides (1000 mg/dL, hyperlipidemia simulation), human serum albumin (HSA, 4.5 g/dL), and rheumatoid factor (RF, 200 IU/mL). Each interferent was tested individually and in combination at fixed Myo (150 ng/mL) or cTnI (50 ng/mL) concentrations (*n* = 3).

All interferents produced < 6% deviation from interferent-free controls, well within CLSI EP07-A2 acceptability criteria (± 10% bias). The combined 10% BSA blocking and Tween-20 sample pad pretreatment minimized non-specific adsorption, while covalent AuNPs@Ab conjugation maintained probe integrity in high-protein/lipid matrices. These results demonstrate superior matrix tolerance compared to physisorbed commercial LFIA, supporting reliable quantification in hemolyzed, icteric, or lipemic clinical specimens.

### Performance analysis based on gold nanoparticles

**Fig. 3:**
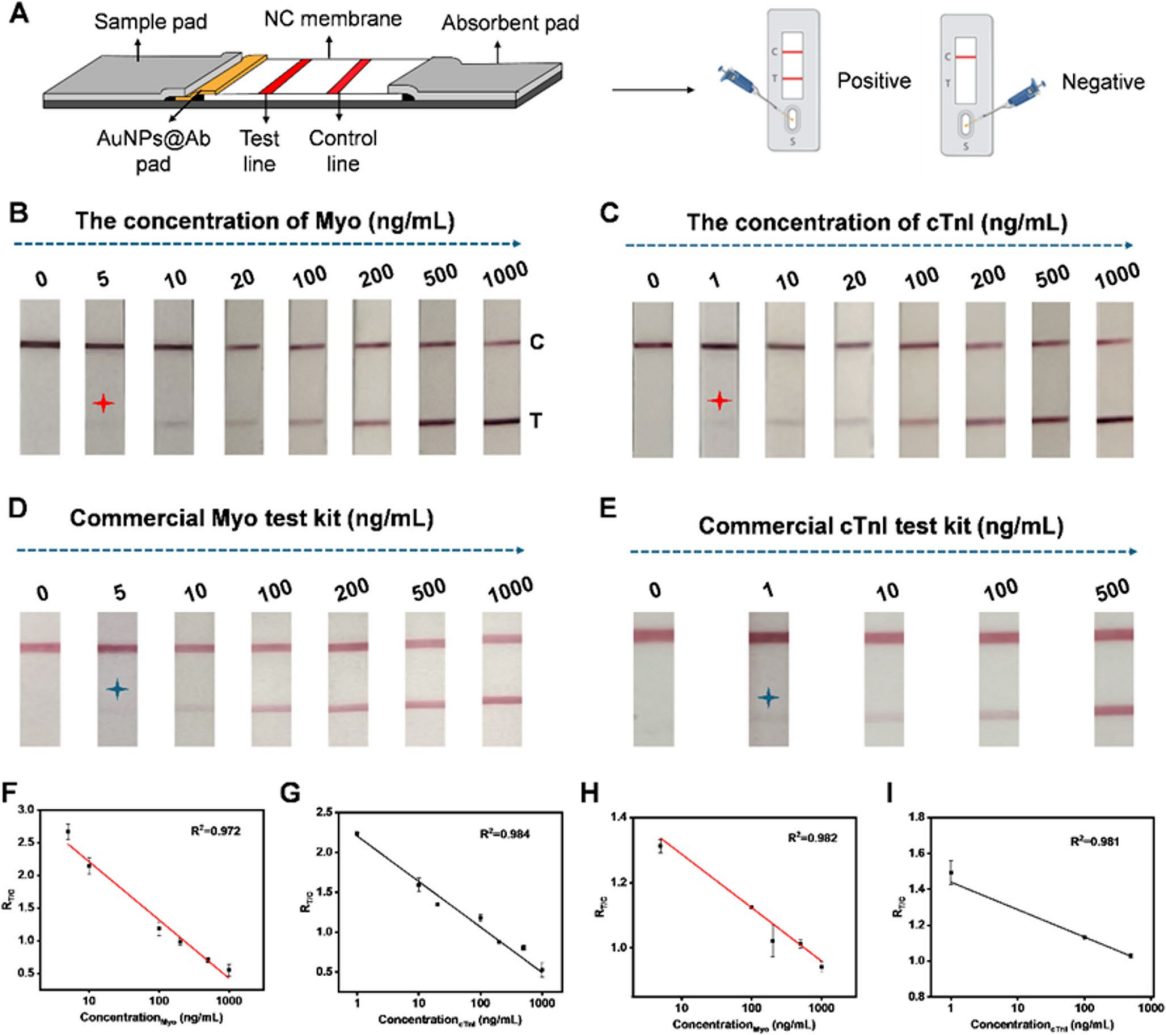
Detection of Myo and cTnI using AuNP-based LFA. (**A**) Schematic illustration of the LFA configuration and representative detection outcomes. (**B**) Readout results of AuNP-based LFA for varying concentrations of Myo. (**C**) Readout results of AuNP-based LFA for varying concentrations of cTnI. (**D**) Readout results of a commercial LFA for different concentrations of Myo. (**E**) Readout results of a commercial LFA for different concentrations of cTnI. (**F**) Calibration curve showing the linear relationship between Myo concentration and corresponding signal intensity. (**G**) Calibration curve showing the linear relationship between cTnI concentration and corresponding signal intensity. (**H**) Calibration curve of Myo obtained from the commercial LFA. (**I**) Calibration curve of cTnI obtained from the commercial LFA. Data are presented as mean ± SD (*n* = 3)

LFIA enables rapid target detection via capillary action across five components: sample pad, conjugate pad, NC membrane, absorbent pad, and PVC backing (Fig. 1A). As illustrated in Fig. 1C, our AI-enhanced workflow provides quantitative readout at 8 min vs. commercial LFIA’s 15-min visual endpoint, while covalent AuNPs@Ab conjugation yields 3–5× signal enhancement (Fig. 3B vs. 3D).The sample pad serves to receive the test sample, while the conjugate pad is preloaded with AuNPs@Ab probes. The NC membrane is marked with T line and C line for target identification and assay validation. The absorbent pad maintains the capillary flow of the liquid (Fig. 3A). To achieve optimal detection sensitivity and ensure consistent performance in practical applications, we first systematically optimized the detection conditions. Variables included the volume of AuNPs@Ab probes, the applied sample amount, and the concentration of capture antibodies on the NC membrane. Under a fixed antigen concentration, the detection performance was evaluated by comparing the signal intensity ratio of the T and C lines (T/C). Taking Myo as a model analyte, the optimal conditions were determined to be 1 µL AuNPs@Ab, 60 µL sample volume, and a capture antibody concentration of 1 mg/mL, whereas for cTnI, the optimal conditions were 1 µL AuNPs@Ab, 50 µL sample volume, and 0.8 mg/mL capture antibody (Figure S1-S2).

Under optimized detection conditions, we further evaluated the performance of the platform for both biomarkers. For Myo (5-1000 ng/mL), T-line R-channel intensity decreased proportionally with concentration in the competitive sandwich format, where excess antigen saturated capture sites and reduced accumulated AuNPs@Ab (Fig. 3B). T/C ratios exhibited strong linearity: y = -0.892 log₁₀(x) + 3.10 (R² = 0.972) across 5-1000 ng/mL (Fig. 3F), confirming the inverse correlation characteristic of competitive LFIA. Visual LOD = 5 ng/mL. For cTnI (1-1000 ng/mL), T-line intensity similarly decreased following the identical competitive mechanism (Fig. 3C). Quantitative analysis was performed using ImageJ to extract the R-channel value of both the T and C lines, followed by calculation of the T/C ratio (R_T/C_) and curve fitting against concentration. T/C calibration showed: y = -0.570 log₁₀(x) + 2.21 (R² = 0.984) (Fig. 3G); visual LOD = 1 ng/mL.

To further compare the performance of functionalized AuNPs-LFIA with that of commercial AuNPs-LFIA, we tested Myo and cTnI using commercially available LFA strips. In the commercial LFAs, the detection signals (R-channel) of Myo and cTnI decreased with increasing concentrations (Fig. 3D-E). The linear regression equations of y = -0.164 Lg(x) + 1.45 (R^2^ = 0.982) and y = -0.153 Lg(x) + 1.44 (R^2^ = 0.981), respectively, indicating a good linear correlation between signal intensity and analyte concentration (Figure S4, Fig. 3H-I). As shown by comparing Fig. 3B with Fig. 3D, the variation in R_T_ values observed in functionalized AuNPs-LFIA was markedly greater than that of the commercial counterparts. These findings indicate that antibody-functionalized AuNPs substantially enhance the specific binding efficiency to the target, thereby improving colorimetric signal sensitivity and resolution.

Both analytes exhibited identical inverse T/C trends characteristic of the competitive sandwich format, where increasing antigen saturates fixed capture Ab sites, reducing probe accumulation at T-lines. Minor slope differences (-0.892 vs. -0.570) reflect antibody affinity variations but maintain consistent quantitative behavior across biomarkers.

**Fig. 4:**
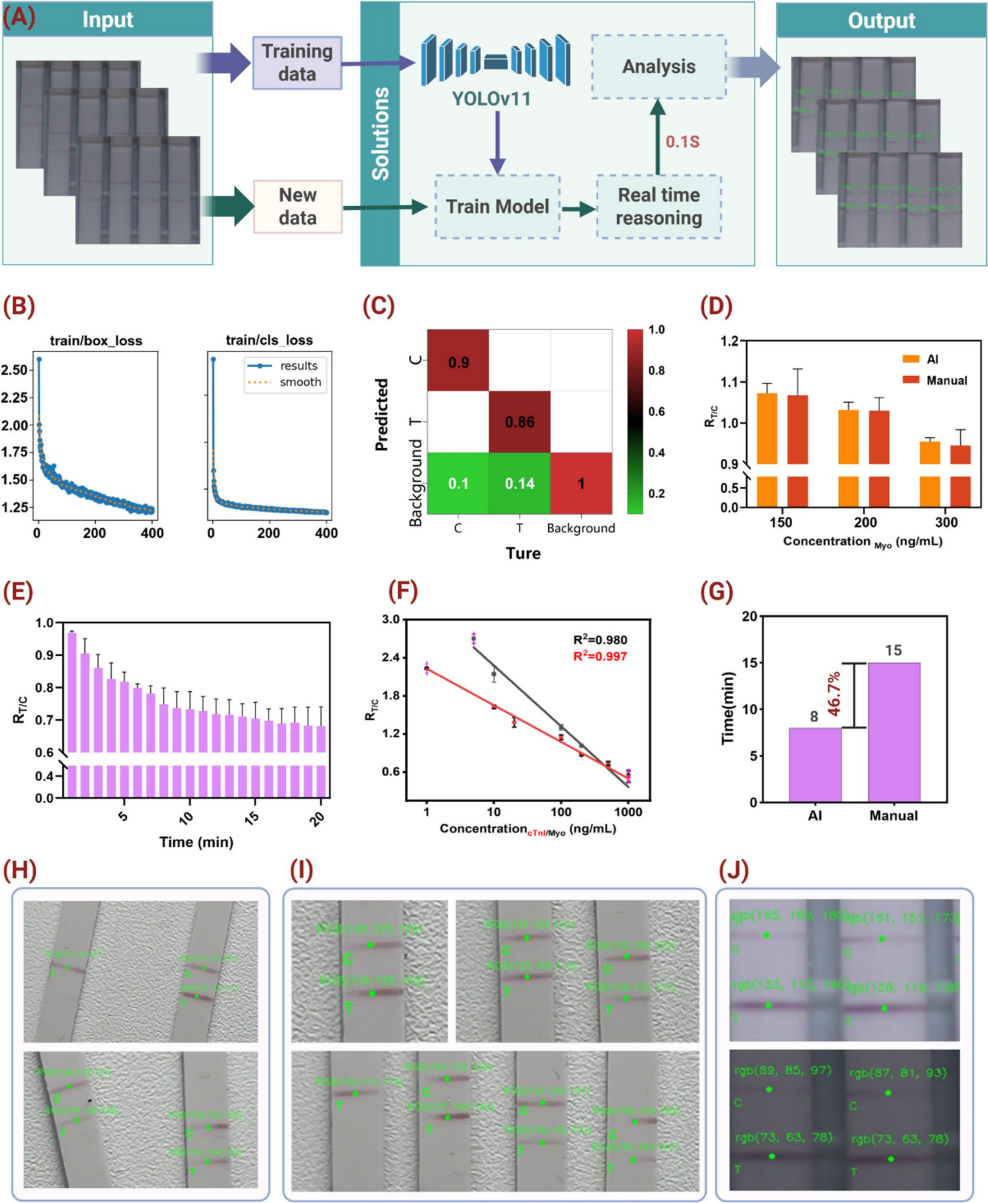
Machine vision-based analysis of LFIA. (**A**) Schematic workflow of machine vision-based detection of the T and C lines on LFIA. (**B**) Loss curves during the training of the machine vision model, including bounding box localization loss and classification loss. (**C**) Normalized confusion matrix of the model’s classification results, showing the accuracy of identifying different regions. (**D**) Statistical comparison of T/C ratios in the R channel obtained by machine vision versus manual analysis at Myo concentrations of 150, 200, and 300 ng/mL. (**E**) Dynamic change of the R_T/C_ value over time at a Myo concentration of 300 ng/mL. (**F**) Standard curves for cTnI and Myo generated based on machine vision detection results. (**G**) Comparison of analysis time between machine vision and manual detection, with the former saving approximately 7 min per test. (**H**-**J**) Model generalizability tests under varying positions, angles, quantities, and ambient lighting conditions

The uniform inverse correlation simplifies machine vision calibration (Fig. 4F) and eliminates configuration-specific interpretation errors, while our covalent AuNPs@Ab deliver 3–5× steeper slopes than physisorbed commercial LFIA (Fig. 3H, I), confirming enhanced analytical sensitivity. To further contextualize these performance improvements, we compared the overall analytical capabilities of the developed platform with those of conventional commercial LFIA systems.

### Innovation and comparative advantages

To further evaluate the performance of the developed platform, we compared the AI-assisted LFIA with conventional commercial LFIA systems in terms of sensitivity, assay time, and analytical robustness.

First, the use of covalently functionalized AuNPs@Ab probes resulted in improved analytical sensitivity. Compared with commercial LFIA systems that typically rely on physically adsorbed antibodies, the proposed approach demonstrated stronger and more stable signal generation, as evidenced by the increased T-line intensity (Fig. 3B vs. Figure 3D). This improvement is reflected in the lower limits of detection (LOD) achieved for Myo (0.224 ng/mL) and cTnI (0.071 ng/mL), compared to approximately 5 ng/mL and 1 ng/mL, respectively, for conventional LFIA. The enhanced performance can be attributed to improved antibody stability and reduced non-specific interactions, which are consistent with the observed surface charge shift following conjugation.

Second, the integration of the YOLOv11-based detection model enabled earlier quantitative readout. While conventional LFIA typically requires approximately 15 min for full color development and visual interpretation, the proposed system achieved stable quantitative results at 8 min. As shown in Fig. 4E, the T/C ratio reached a plateau prior to complete visual development, allowing reliable early-stage detection. The strong correlation with standard analysis methods (R² = 0.99) suggests that this reduced assay time does not significantly compromise accuracy. This reduction in time-to-result may be beneficial in time-sensitive clinical scenarios such as acute myocardial infarction (AMI) triage.

Third, the proposed system reduces operator-dependent variability. Manual image analysis methods (e.g., ImageJ) are sensitive to variations in region-of-interest selection and environmental conditions. In contrast, the AI-based approach demonstrated high consistency across varying imaging conditions (Fig. 4H–J), with a maximum deviation of 1.38% and an overall agreement of 99.16% with manual measurements. These results indicate improved robustness and reproducibility, which are important for point-of-care applications.

A summary of the comparative performance is provided in Table 1. Overall, the developed platform demonstrated lower detection limits, shorter assay time, and reduced variability compared with conventional LFIA systems.

**Table 1:** Quantitative comparison between conventional LFIA and the proposed AI-assisted LFIA platform

Metric	Commercial LFIA	Our AI-LFIA
Myo LOD	~ 5 ng/mL	0.224 ng/mL
cTnI LOD	~ 1 ng/mL	0.071 ng/mL
Time-to-Result	15 min	8 min
Operator Variability	High (visual)	< 1.4% (AI)
R² vs. Reference	0.98	0.996

Note: Data represent limits of detection (*LOD*), time-to-result, operator variability, and correlation coefficients (*R²*) versus reference methods(*n = 3 replicates*)*. *The AI-LFIA demonstrates 22–70× lower LODs, 46.7% faster readouts, and < 1.5% variability, enabling reliable point-of-care deployment by non-experts

Taken together, the results suggest that the combination of improved probe chemistry and AI-assisted analysis can enhance the analytical performance and usability of LFIA systems, while further studies are needed to confirm its clinical applicability in real-world settings. Following this comparative analysis, we further evaluated the system’s performance using machine vision-based quantitative analysis.

Machine Vision-Based Quantitative Analysis A real-time LFIA analysis system was developed using YOLOv11 for simultaneous T/C line detection across multiple strips. Hardware comprised a Basler ace acA1920-155 μm CCD camera (12 MP, > 70 dB SNR) and NVIDIA Jetson Orin Nano edge computer. Custom Python software (OpenCV 4.8.1, NumPy 1.26, Pillow 10.1) orchestrated image acquisition (5 fps), inference, and T/C ratio computation. Post-detection, R-channel means were extracted from bounding boxes using non-max suppression (confidence threshold > 0.6, IoU threshold = 0.45). The T-line R-channel intensity (R_T) was calculated as the mean RGB value within the T-line bounding box, while the C-line R-channel intensity (R_C) was similarly computed from the C-line bounding box. The normalized T/C ratio was then determined using the formula T/C = (255 - R_T) / (255 - R_C), which accounts for background illumination variations and provides a stable quantitative metric. The complete workflow (Fig. 4A) achieved end-to-end latency of 120 ms per image, enabling 8-minute quantitative readouts versus 15-minute manual inspection. During training, both the bounding box loss and classification loss steadily decreased and converged around the 400th epoch, indicating robust model performance (Fig. 4B, Figure S5). Figure 4C presents the model’s classification performance for C- and T-line recognition (Figure S6-S7). The normalized confusion matrix shows an accuracy of 90% for C-line detection and 86% for T-line detection, with minimal misclassification of background regions. Most errors mainly occurred at the early color-development stage of T and C lines, when the strip coloration was not fully developed.

To assess detection accuracy, Myo concentrations of 150, 200, and 300 ng/mL were measured in triplicate by both machine vision detection and manual colorimetric measurement. Results from the two approaches were highly consistent (Fig. 4D), with an average agreement of 99.16%. The maximum error was 1.38% for machine vision and 4.63% for manual detection, indicating that machine vision reduced errors by approximately 3.35-fold and substantially improved the quantitative analysis reliability.

Furthermore, to assess dynamic detection performance, the R-channel T/C ratio was monitored over time at a Myo concentration of 300 ng/mL (Fig. 4E). The T/C ratio gradually decreased with required readout time, reaching a plateau at ~ 8 min and stabilizing by 15 min. Thus, the 8-minute time point was selected as the final detection node to balance sensitivity and efficiency.

Figure 4F shows the standard calibration curves for cTnI and Myo obtained via machine vision. The cTnI linear equation was y = -0.955Lg(x) + 3.23 with R² = 0.997, y = -0.577Lg(x) + 2.23 with R² = 0.980, demonstrating strong linear responses and validating the method’s applicability across multiple targets. The limits of detection (LOD) for Myo and cTnI were calculated using the formula LOD = 3δ/S, where δ represents the standard deviation of the low-concentration group and S denotes the slope of the calibration curve, yielding values of 0.224 ng/mL and 0.071 ng/mL, respectively. A comparison of detection time between machine vision and manual measurement (Fig. 4G) showed that automated detection reduced the average assay time by 7 min, improving efficiency by ~ 46.7%, making it highly suitable for point-of-care diagnostics and large-scale screening.

Finally, to evaluate model generalizability, tests were conducted under varying strip positions, orientations, quantities, and ambient lighting conditions. As shown in Figs. 4H-J, the system maintained robust performance under these complex conditions, underscoring its strong adaptability and broad applicability of the proposed machine vision model.

**Fig. 5:**
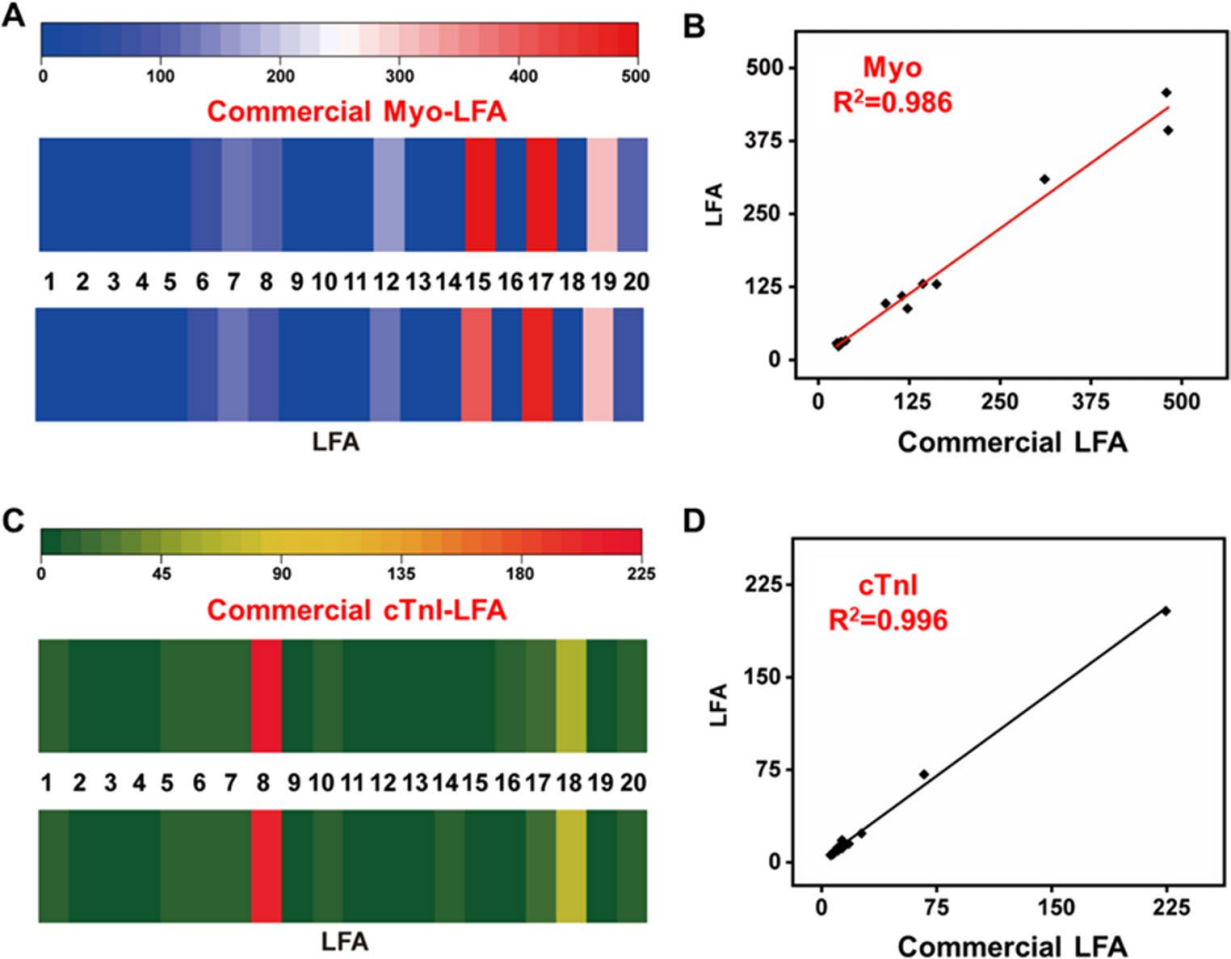
Comparative analysis of spiked Myo and cTnI samples using the AuNPs-LFIA platform and commercial ELISA kits. (**A**) Comparison of Myo detection results at different spiked concentrations, showing good agreement between the AuNPs-LFIA platform and commercial LFIA. (**B**) Correlation analysis between Myo concentrations measured by the AuNPs-LFIA platform and commercial LFIA, with a correlation coefficient of R² = 0.986. (**C**) Comparison of cTnI detection results in spiked samples between the AuNPs-LFIA platform and commercial LFA. (**D**) Correlation analysis of cTnI detection results between the AuNPs-LFIA platform and commercial LFA, demonstrating a high degree of consistency

To further evaluate the reliability and practical applicability of the AuNPs-LFIA platform integrated with the YOLOv11 deep learning algorithm for clinical sample analysis, a series of spiked experiments were designed to simulate real clinical conditions. Specifically, Myo and cTnI, two key biomarkers of myocardial injury, were spiked into FBS to generate a set of test samples. These samples were then analyzed using both the developed AuNPs-LFIA platform and commercial assay kits, and the detection performance and consistency of the two methods were compared across different concentration levels. As shown in Fig. 5A and C, the AuNPs-LFIA platform exhibited a clear concentration-dependent response for both Myo and cTnI, with R-channel signal intensity decreasing as analyte concentration increased. Notably, when combined with the YOLOv11 model, the LFA platform was capable of automatically identifying the T and C lines and accurately calculating the T/C ratio, significantly enhancing image recognition accuracy and data processing efficiency. Even at low analyte concentrations, the platform maintained high sensitivity and discriminative capability, a critical feature for the early detection of myocardial injury. To further assess the concordance between the LFA platform and commercialized LFIA, regression analysis was performed on the results obtained by the two methods, and correlation curves were plotted (Fig. 5B and D). The Myo detection data showed an excellent fit with a correlation coefficient (R²) of 0.986, indicating high agreement between the methods; similarly, the R² for cTnI was 0.996, confirming the platform’s strong consistency and broad applicability across different myocardial injury biomarkers. Beyond analytical performance, the AuNPs-LFIA combined with YOLOv11 offered significant advantages in workflow simplicity, response speed, and operational convenience. The lateral flow design enabled rapid antigen-antibody interaction and signal generation with only a single droplet of sample applied to the sample pad, completing the entire assay within 8 min. Simultaneously, the YOLOv11 algorithm provided real-time image recognition and quantitative analysis of the T line, eliminating subjective errors associated with manual interpretation and markedly improving result objectivity and reproducibility.

Despite the promising performance of the AuNPs-LFIA platform integrated with machine vision, several limitations should be noted to support accurate interpretation of the results and guide future improvements.

First, the evaluation of the system was conducted exclusively using spiked FBS samples rather than clinical human specimens, which may not fully represent the complexity of real patient samples. Factors such as variable protein composition, endogenous interferents, hemolysis, lipemia, and patient-to-patient variability could influence assay performance. Consequently, the current results demonstrate analytical feasibility but not clinical diagnostic validity.

Second, the study did not assess long-term storage stability, batch-to-batch reproducibility, or durability of AuNPs@Ab conjugates and LFA strips. These metrics are essential for translation toward real-world point-of-care (POC) deployment.

Third, although the machine vision model demonstrated strong performance, its accuracy was evaluated on a relatively limited dataset (750 images), and additional large-scale datasets with broader variability in lighting, camera types, and strip appearance would further strengthen the model’s robustness.

Finally, the comparison with commercial LFIA kits focused only on correlation analysis, without access to standardized clinical benchmarks or professionally validated diagnostic kits. Therefore, while the high R² values indicate good analytical agreement, the comparison cannot substitute for regulatory-grade validation.

Future work should include clinical testing on human samples, extended environmental stress testing, evaluation of operational robustness in real emergency settings, and comprehensive comparisons with certified diagnostic tools.

## Conclusion

In this study, we developed a LFIA platform (AuNPs-LFIA) that integrates functionalized (AuNPs) with machine vision, enabling rapid, sensitive, and quantitative detection of cardiac injury biomarkers Myo and cTnI. The platform demonstrated detection limits of 0.224 ng/mL for Myo and 0.071 ng/mL for cTnI, with results showing strong concordance with commercial assay kits (R² > 0.95), confirming its analytical reliability. Moreover, the integration of deep learning-assisted machine vision substantially reduced the detection time, shortening traditional 15-minute visual qualitative readouts to just 8 min for quantitative analysis, while effectively eliminating subjectivity associated with manual interpretation. This advancement has the potential to significantly enhance the POCT, supporting both healthcare professionals and non-expert operators to make efficient and accurate clinical decisions. Looking forward, this strategy offers broad applicability and could be extended to the detection of additional disease-related biomarkers, such as those relevant for influenza and other infectious diseases, facilitating rapid on-site diagnostics.

The reduction of readout time from 15 min to an optimal 8-minute detection node highlights the practicality of combining nanotechnology with machine vision for accelerating decision-making. It should be emphasized that the chemical reaction time of LFIA remains unchanged; rather, the machine-vision system enables earlier and more objective interpretation of partially developed test and control lines, improving operational efficiency in emergency or resource-limited settings.

Furthermore, the AI-assisted platform provides consistent quantitative metrics that minimize user subjectivity, making it suitable for deployment by non-specialist personnel such as paramedics, community health workers, and first responders. These features collectively indicate strong potential for integrating the system into pre-hospital AMI screening workflows.

Future work will focus on validating the platform using clinical human serum samples, performing long-term stability studies for both AuNPs@Ab conjugates and LFIA strips, and expanding the machine-vision dataset to include broader real-world imaging conditions. Integration with mobile-device cameras and development of a portable smartphone-based reader will also be explored to enhance accessibility. Additionally, extending this approach to other clinically relevant biomarkers could establish a versatile and scalable POCT system.

## Electronic Supplementary Material

Below is the link to the electronic supplementary material.

Supplementary Material 1 (DOCX 504 KB)

## Data Availability

All data supporting the findings of this study are available within the paper and its Supplementary Information.
